# Common Genetic Variant in VIT Is Associated with Human Brain Asymmetry

**DOI:** 10.3389/fnhum.2016.00236

**Published:** 2016-05-24

**Authors:** Sayed H. Tadayon, Maryam Vaziri-Pashkam, Pegah Kahali, Mitra Ansari Dezfouli, Abdolhossein Abbassian

**Affiliations:** ^1^School of Cognitive Sciences, Institute for Research in Fundamental SciencesTehran, Iran; ^2^School of Mathematics, Institute for Research in Fundamental SciencesTehran, Iran; ^3^Vision Sciences Laboratory, Department of Psychology, Harvard UniversityCambridge, MA, USA; ^4^Department of Cell and Molecular Biology, School of Biology, College of Science, University of TehranTehran, Iran

**Keywords:** asymmetry, genome-wide association study, surface area, lateralization, imaging genetics

## Abstract

Brain asymmetry varies across individuals. However, genetic factors contributing to this normal variation are largely unknown. Here we studied variation of cortical surface area asymmetry in a large sample of subjects. We performed principal component analysis (PCA) to capture correlated asymmetry variation across cortical regions. We found that caudal and rostral anterior cingulate together account for a substantial part of asymmetry variation among individuals. To find SNPs associated with this subset of brain asymmetry variation we performed a genome-wide association study followed by replication in an independent cohort. We identified one SNP (rs11691187) that had genome-wide significant association (*P*_*Combined*_ = 2.40e-08). The rs11691187 is in the first intron of *VIT*. In a follow-up analysis, we found that *VIT* gene expression is associated with brain asymmetry in six donors of the Allen Human Brain Atlas. Based on these findings we suggest that *VIT* contributes to normal brain asymmetry variation. Our results can shed light on disorders associated with altered brain asymmetry.

## Introduction

Like many other biological systems, human brain shows structural asymmetry (Geschwind and Galaburda, 1985; Corballis, 2014). However, mechanisms underlying brain asymmetry are largely unknown (Bishop, 2013). The contribution of genetic factors in brain asymmetry has been a matter of debate. Recent imaging genetic studies have found genetic factors that contribute to brain asymmetry and asymmetry variation. In a GWAS study, Guadalupe et al. (2015) reported that genes involved in steroid hormone biology might influence population variance in planum temporale asymmetry. In a twin study, Jahanshad et al. (2010) showed that genetic factors have moderate effect in accounting for asymmetry variance in several white matter tracts. On the other hand, in a twin study Eyler et al. (2014) found high genetic correlations between the two hemispheres for cortical surface and thickness and thus suggested that genetic factors might play low to moderate role in asymmetry between two hemispheres. In all likelihood no single gene can account for all observed brain asymmetry variation. In a study of asymmetries in brain's intrinsic activity fluctuations at rest, Liu et al. (2009) showed that multiple independent factors capture the variation in brain asymmetry. This suggests that a single gene might only explain a subset of brain asymmetry variation across individuals.

Recently, large-scale genetic studies have been combined with brain imaging to unravel genetic variants associated with anatomical and functional brain characteristics (Marenco and Radulescu, 2010; Bakken et al., 2012; Stein et al., 2012; Jahanshad et al., 2013; Cai et al., 2014; Roussotte et al., 2014; Ramanan et al., 2015) and neurological and neuropsychiatric disorders (Callicott et al., 2005; Potkin et al., 2009). In this study, we aimed to find genetic correlates associated with variation of cortical surface area asymmetry among individuals. Anatomical brain asymmetry can be measured using morphological features such as cortical volume, cortical thickness and cortical surface area (Hutsler et al., 1998; Luders et al., 2006; Lyttelton et al., 2009; Koelkebeck et al., 2014; Meyer et al., 2014; Takaya et al., 2015). We used cortical surface area asymmetry as it has been shown that brain asymmetry is more prominent in cortical surface area (Koelkebeck et al., 2014). We computed lateralization index for 34 cortical regions across the cerebral cortex. Distinct brain regions might covary in structural brain asymmetry and it is reasonable to presume that increased asymmetry of one area might be accompanied by increased or decreased asymmetry of other areas. Genetic factors that modulate brain asymmetry in one region might influence extent of brain asymmetry in other regions. Therefore, instead of using brain asymmetry measures from individual regions, we performed principal component analysis (PCA) to compute an asymmetry measure that captures most variance in brain asymmetry across individuals. After identifying the component that explained a substantial amount of asymmetry variation, we designed a two-stage genome wide association study (GWAS; Satagopan et al., 2004; Skol et al., 2006). In the first phase, we performed a GWAS to find SNPs that were associated with this asymmetry measure. Subsequently, we tested our top SNP and were able to replicate our finding in a separate dataset.

## Methods

### Subjects

In order to study brain asymmetry variation among subjects, we studied 706 right-handed subjects [Healthy controls (CN) = 209, Late Mild Cognitive Impairment (LMCI) = 320, Alzheimer's Disease (AD) = 177] from ADNI-1 cohort. To replicate PC1 and its reliability across time, we used 119 healthy participants from ADNI-2/GO that had been imaged at four different time points (screening time, month 6, 12, 24). For GWAS, we restricted our study to non-Hispanic Caucasian subjects. We replicated our top SNP in an independent population of non-Hispanic Caucasian right-handed participants that had been genotyped in ADNI-2/GO cohort (CN = 108, Early-Mild Cognitive Impairment (EMCI) = 168, LMCI = 55).

The ADNI was launched in 2003 by the National Institute on Aging (NIA), the National Institute of Biomedical Imaging and Bioengineering (NIBIB), the Food and Drug Administration (FDA), private pharmaceutical companies, and non-profit organizations, as a $60 million, 5-year public- private partnership. The primary goal of ADNI has been to test whether serial magnetic resonance imaging (MRI), positron emission tomography (PET), other biological markers, and clinical and neuropsychological assessment can be combined to measure the progression of mild cognitive impairment (MCI) and early AD. Determination of sensitive and specific markers of very early AD progression is critical to aid researchers and clinicians to develop new treatments and monitor their effectiveness, and to reduce the time and cost of clinical trials. The Principal Investigator of this initiative is Michael, W. Weiner, M. D, VA Medical Center and University of California, San Francisco. ADNI is the result of efforts of many co-investigators from a broad range of academic institutions and private corporations, and subjects have been recruited from over 50 sites across the U.S. and Canada. The initial goal of ADNI was to recruit 800 subjects but ADNI has been followed by ADNI-GO and ADNI-2. To date these three protocols have recruited over 1500 adults, ages 55–90, to participate in the research, consisting of cognitively normal older individuals, people with early or late MCI (EMCI or LMCI), and people with early AD. The follow up duration of each group is specified in the protocols for ADNI-1, ADNI-2, and ADNI-GO. Subjects originally recruited for ADNI-1 and ADNI-GO had the option to be followed in ADNI-2. Thousands of longitudinal imaging scans, performance on neuropsychological and clinical assessments, and biological samples were collected at baseline and at follow-up visits for all or a subset of participants. Genome-wide genotyping data are available on the full ADNI sample. For up-to-date information, see www.adni-info.org.

### Genotyping

Genome-wide genotype data were collected using the Illumina Human610-Quad BeadChip (620901 markers) for ADNI-1 cohort. 505853 autosomal SNPs and 490 subjects (CN = 155, LMCI = 210, AD = 125) passed quality control filters (sample call rate > 95%, SNP call rate > 99%, minor allele frequency > 5%, Hardy–Weinberg disequilibrium *P* < 1e-6). For replication study, we studied 331 non-Hispanic Caucasian subjects from ADNI2/GO cohort who had been genotyped using the Illumina HumanOmniExpress BeadChip (730525 markers). 599425 SNPs and 330 subjects passed quality control filters same as above.

### Image acquisition

High-resolution structural brain MR images from the baseline visit were collected from ADNI-1 cohort. Structural MRI scans in the ADNI-1 study were obtained using a standardized protocol to maximize consistency across 58 image acquisition sites, using 1.5 Tesla MRI scanners. A T1-weighted 3D MPRAGE sequence was used (TR/TE = 2400/1000 ms; flip angle = 8°; FOV = 24 cm; with a final voxel resolution = 0.9375^*^0.9375^*^1.2 mm3). We also used processed images of ADNI-2/GO subjects downloaded from ida.loni.usc.edu.

### Cortical surface area and lateralization index

Cortical reconstruction was performed with the Freesurfer image analysis suite (Version 5.3.0), which is documented and freely available for download online (http://surfer.nmr.mgh.harvard.edu/). In brief, the processing stream includes a Talairach transform of each participant's native brain, removal of non-brain tissue, and segmentation of gray matter (GM)/white matter (WM) tissue. The GM/WM boundary was tessellated to generate multiple vertices across the whole brain. The cortical surface of each hemisphere was inflated to a sphere to locate the pial surface and the GM/WM boundary. After the creation of cortical representations, all vertices were assigned neuroanatomical labels on a cortical surface model based on the automated labeling system, and the entire cortex of each hemisphere was parcellated into 34 brain regions based on Desikan-Killiany atlas (Desikan et al., 2006). The inner surface area of a region was computed by summing up the area of the vertices in that region. After calculating the cortical surface area for each brain region and each hemisphere, the lateralization index was computed as a standardized asymmetry index (Left region – Right region)/(Left region + Right region).

### Statistical analysis and genome-wide association study

Statistical analyses were performed using R and Python. We performed PCA after centering asymmetry scores for each region without scaling. For first part of our study, we performed PCA in CN, LMCI, and AD group separately. We used the first principal component (PC1) for our later analysis. To compare PC1 across groups, we ran two different analyses. In the first analysis, we calculated the correlation between the PC1 loadings from the CN group with the PC1 from the AD and LMCI groups. In the second analysis, we projected the individual asymmetry scores from the LMCI and AD groups onto the PC1 of the CN group to calculate projected PC1 (PC1_*p*_) for each individual. We then correlated the PC1_*p*_ scores with the original PC1 scores from each group. As PC1 was highly similar between groups, we used PC1 from CN group as the reference axis to compute PC1 scores for subsequent analyses.

We tested each SNP for association using PLINK (Purcell et al., 2007) to fit an additive linear model with minor allele count, age, gender, and diagnosis as predictors of PC1 score. Genomic inflation (λ_*GC*_) was estimated in the standard way by dividing the median observed χ2 statistic from the GWAS by 0.456, the approximate median of a χ2 distribution with one degree of freedom (Devlin et al., 2001). We tested association of our top SNP (rs11691187) in an independent dataset fitting a same linear model.

### Allen Human Brain Atlas

The Allen Human Brain Atlas (AHBA) is a publicly available online resource of gene expression information in the adult human brain (Allen Institute for Brain Science^1^; Hawrylycz et al., 2012). To construct the AHBA, samples were collected from 6 donors, four of whom only donated their left hemispheres and two donated both hemispheres. Approximately 500 samples were collected per hemisphere. To enable comparison across brains, microarray normalization was applied to the gene expression values both within and across brains (Allen Institute for Brain Science, 2013). All brains underwent structural MRI before dissection, and were normalized to MNI space.

*VIT* was sampled by two probes. We chose the probe whose average expression across all regions was larger (A_23_P56578). Log2 *VIT* gene expressions were obtained for all samples taken from left anterior cingulate cortex. We only focused on the samples from the left hemisphere, as all donors had samples from this hemisphere. We then calculated both mean and median *VIT* gene expression across samples for each individual, and used these values to determine the relationship between the PC1 scores and *VIT* gene expression in left anterior cingulate cortex.

T1-weighted MRIs from all six subjects were analyzed using Freesurfer. Cortical surface areas for 34 regions were extracted as stated previously. Lateralization index was calculated and brain asymmetry scores were projected onto PC1 from CN group to get PC1 score for each subject.

## Results

### Brain asymmetry

We studied asymmetry of cortical surface area in a sample of 706 subjects from ADNI-1 cohort. Subjects were grouped into healthy control (CN), late-mild cognitive impairment (LMCI), and Alzheimer's disease (AD) based on initial evaluation at screening time. For each subject, cortical surface area was measured for 34 regions in each hemisphere. Standardized lateralization index was computed as (left cortical surface area– right cortical surface area)/(left cortical surface area + right cortical surface area).

Similar to previous studies, most brain regions showed significant cortical surface area asymmetry (Koelkebeck et al., 2014; Supplementary Figure 1). Cortical surface area brain asymmetry was compared between three groups. Cortical surface area asymmetry was different between three groups in two areas (frontal pole and pars orbitalis) (One-way ANOVA, *F* < 3.2, *P* < 0.05) but none passed Bonferroni correction (0.05/34 = 0.0014; See Supplementary Table 1).

**Figure 1:**
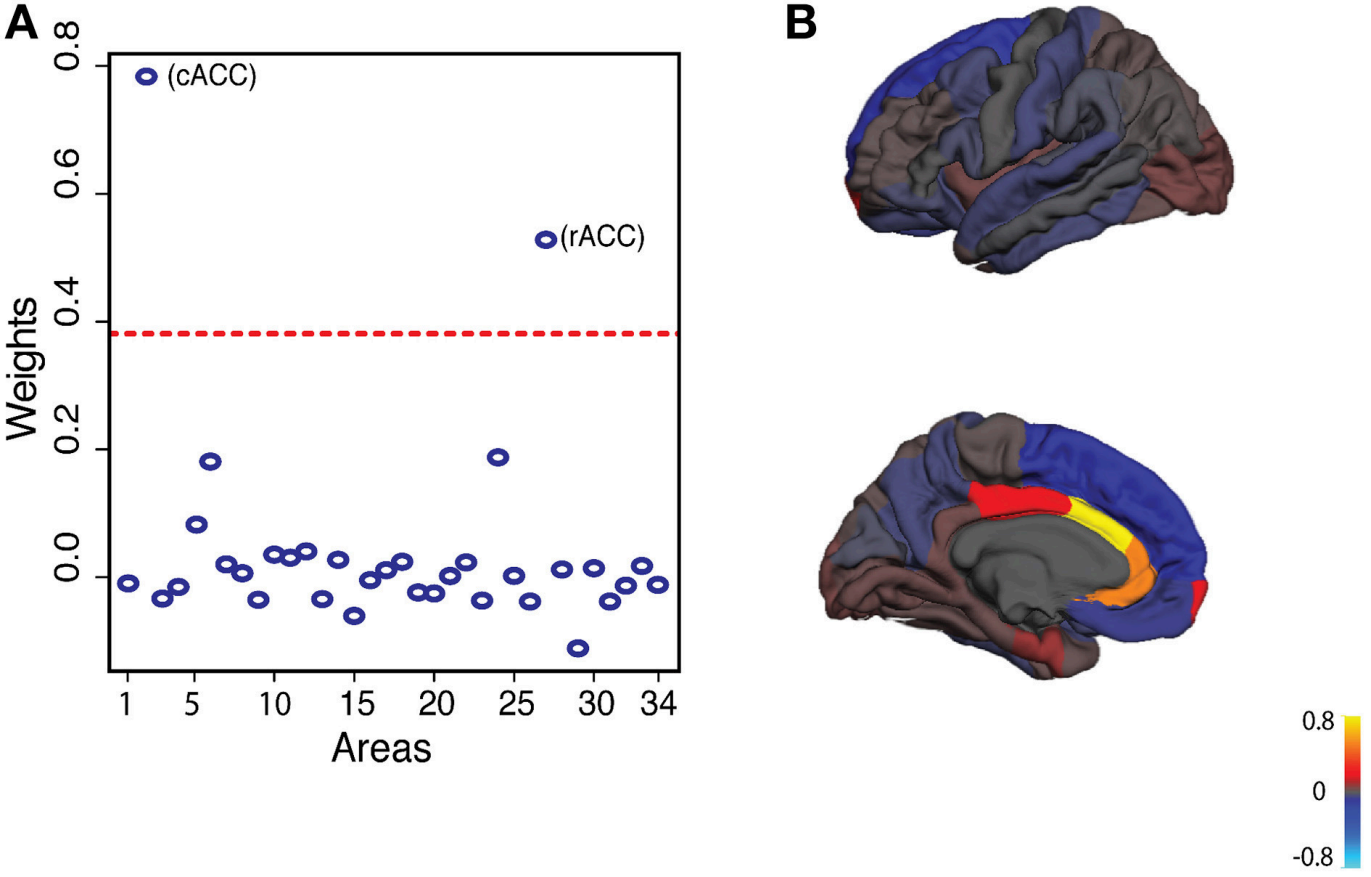
**Principal component analysis of cortical surface area asymmetry in CN group**. **(A)** In all three groups (CN, LMCI, AD), first principal component explains around 13% of variation. First principal component loadings show that areas 2 and 27 (caudal anterior cingulate and rostral anterior cingulate) have the largest weights. Dashed line represents two standard deviations from the mean of weights. **(B)** First principal component loadings on average cortical surface.

We performed PCA on brain asymmetry scores to capture correlated variation for CN, LMCI and AD subjects separately. PCA decomposes brain asymmetry into orthogonal components each accounting for part of observed brain asymmetry variation (Habeck, 2010). The first principal component (PC1) explained ~13% of brain asymmetry variation in all three groups. The first 22 principal components explain around 90% of brain asymmetry variation (Supplementary Figure 2). For the rest of the study, we only focused on PC1 as a subset of brain asymmetry variation among individuals. A more detailed look at the weights of the PC1 loadings showed that caudal anterior cingulate cortex (cACC) and rostral anterior cingulate cortex (rACC) had the largest weights in all three groups (Figure 1). CACC and rACC had rightward and leftward asymmetry, respectively (Supplementary Figure 1; See Supplementary Figure 3 for loadings of first six principal components).

PC1 loadings were highly correlated between groups. PC1 in the CN group was highly correlated with both the AD group (*r* = 0.89) and the LMCI group (*r* = 0.88). Similarly the PC1 from the LMCI group was highly correlated with the AD group (*r* = 0.92). PC1 scores were not different between groups [One way ANOVA, *F*_(2, 703)_ = 1.103, *P* = 0.332]. In another analysis we projected brain asymmetry scores of AD and LMCI individuals onto PC1 of CN group to calculate the new projected PC1 (PC1_*p*_) for the two groups. PC1_*p*_ scores were highly correlated with the original PC1 scores in both AD (*r* = 0.95) and LMCI (*r* = 0.94) groups (Supplementary Figure 4 and see Methods). These analyses confirmed that the PC1 was highly reliable across groups.

**Figure 2:**
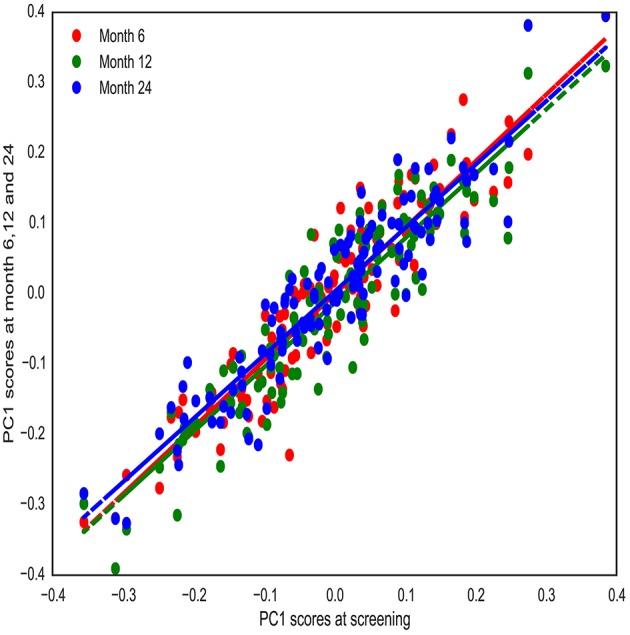
**PC1 stability over time**. PC1 was computed for healthy individuals that have been imaged at four different time points (screening time, month 6, 12, and 24). PC1 scores are highly correlated over time.

We then tested the stability of PC1 over time. To do so, brain asymmetry was computed for another group of healthy participants (from ADNI-2/GO cohort) that had been imaged at 4 different time points [screening time (m0), month 6 (m6), month 12 (m12), and month 24 (m24)] (*n* = 119). Computed PC1 scores at m6, m12, and m24 were highly correlated to PC1 scores at m0 (*r*_*m*0−*m*6_ = 0.92, *r*_*m*0−*m*12_ = 0.91, *r*_*m*0−*m*24_ = 0.92) (see Methods; Figure 2; See Supplementary Figure 5 for stability of all PCs across time). These results confirmed that PC1 was highly stable across groups and time. These tests allowed for combination of the three groups for the subsequent GWAS.

### Genome-wide association study

We hypothesized that normal variation in PC1 scores is partly related to genetic differences between individuals. Therefore, we tested SNPs genome-wide for their association with PC1 scores. Given the stability of PC1 across groups, we combined individuals from the three groups to increase power. We only included non-Hispanic Caucasian participants for GWAS. Particularly, we tested each SNP's strength to predict score of PC1 while controlling for age, gender, and diagnosis.

**Figure 3:**
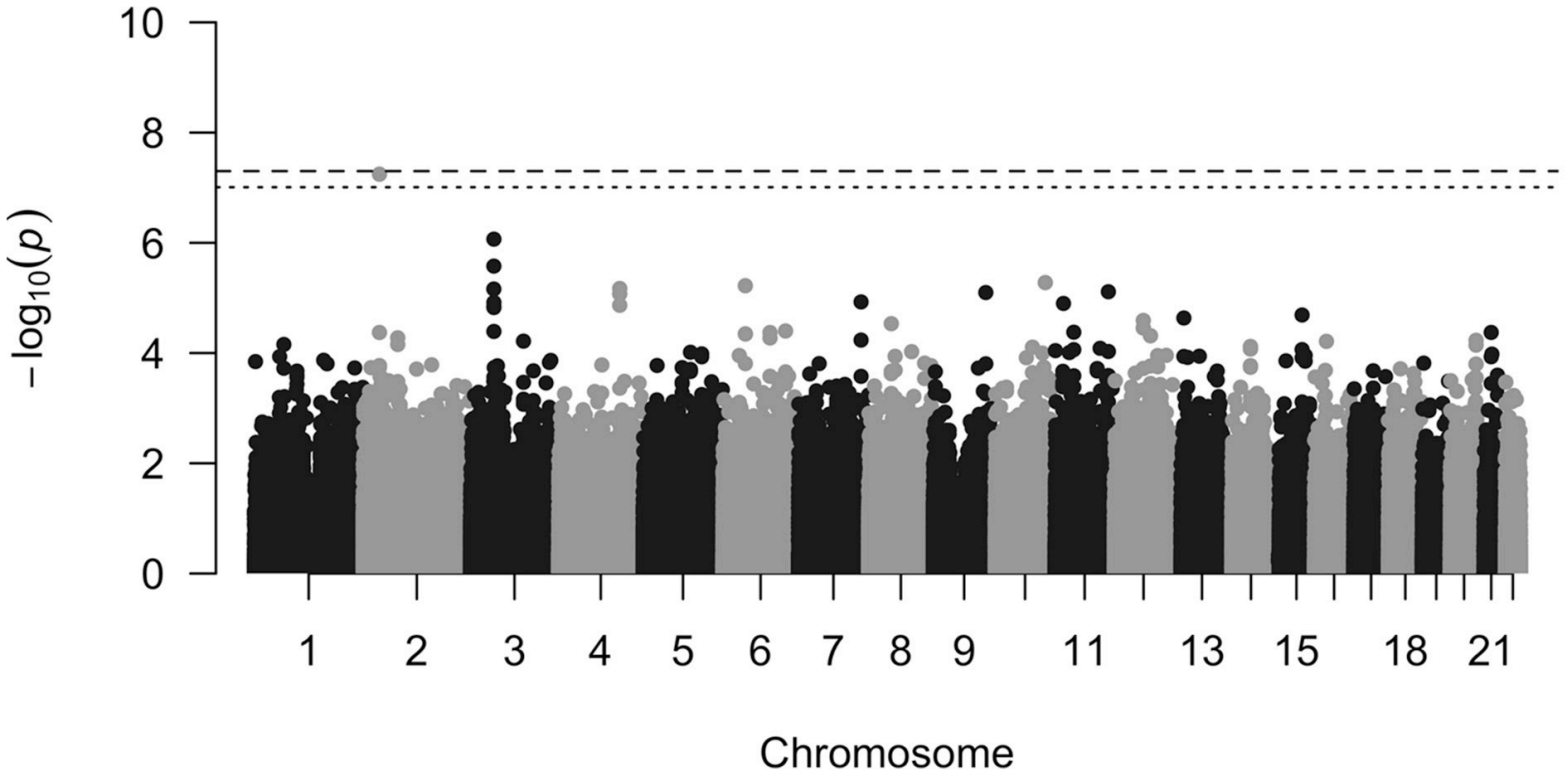
**Manhattan plot**. The dashed line shows genome wide significance threshold and the dotted line shows Bonferroni threshold for this study. The rs11691187 passed Bonferroni threshold and is near genome-wide significance threshold. It passed genome-wide significance threshold after combining with replication dataset (*P* = 2.40e-08).

**Figure 4:**
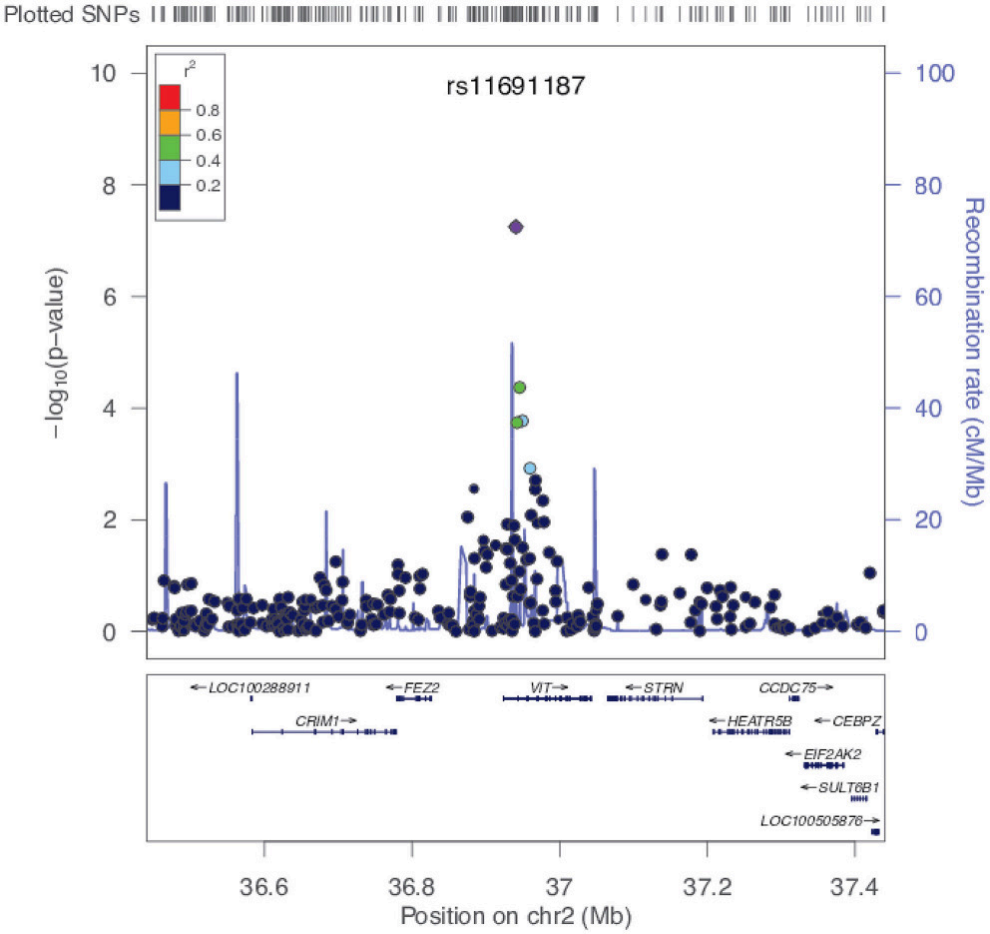
**The rs11691187 regional association plot**. Plot is centered on rs11691187, drawn using LocusZoom software (Pruim et al., 2010). All SNPs are plotted with their association *P*-values against their genomic position. The color of the dots represents the LD between SNPs. The light blue line represents the estimated recombination rates. Genes and exons are shown as dark blue arrows and vertical lines, respectively. The rs11691187 is in the first intron of VIT.

One SNP (rs11691187, minor allele frequency = 0.39) showed strong association with PC1 scores (*P* = 5.69e-8, beta = 0.04511, *SE* = 0.0081) that passed Bonferroni threshold (*P* = 9.8e-8) and reached near genome-wide significance (*P* < 5e-08; Figures 3, 4). Q-Q plot of the distribution of *P*-values showed that the association statistics were approximately normally distributed (Supplementary Figure 6). Genomic inflation (λ_*GC*_) was 1, which indicated that the distribution of *P*-values was unbiased and that the results were not likely to be attributable to population stratification. In the previous analysis, we merged all three groups to increase our sample size. To ascertain that the effect was present with the same trend in the CN group, we tested the association between PC1 and rs11691187 only including the CN subjects (209 subjects). The observed association was in the same direction as our previous analysis (beta = 0.04, *P* = 0.0008).

We then replicated the rs11691187 in an independent dataset. We used 331 right-handed subjects from ADNI-2/GO cohort in which rs11691187 had been genotyped. The subjects were non-Hispanic Caucasian healthy controls or diagnosed with early or late mild cognitive impairment. We tested association of rs11691187 with the computed PC1 scores while controlling for age, gender, and diagnosis (see methods). The rs11691187 was significantly associated with PC1 (*P* = 0.02, beta = 0.0219, *SE* = 0.0095, *n* = 331). No genomic inflation was observed (λ_*GC*_ = 1). We also tested whether the trend could be observed only in CN group (108 subjects). The observed association was in the same direction as our previous analysis (beta = 0.02, *P* = 0.10). The combined *P*-value for rs11691187 reached genome-wide significance (*P*_*combined*_ = 2.40e-08) based on inverse variance-weighted *z*-score (de Bakker et al., 2008).

### Allen Human Brain Atlas

**Figure 5:**
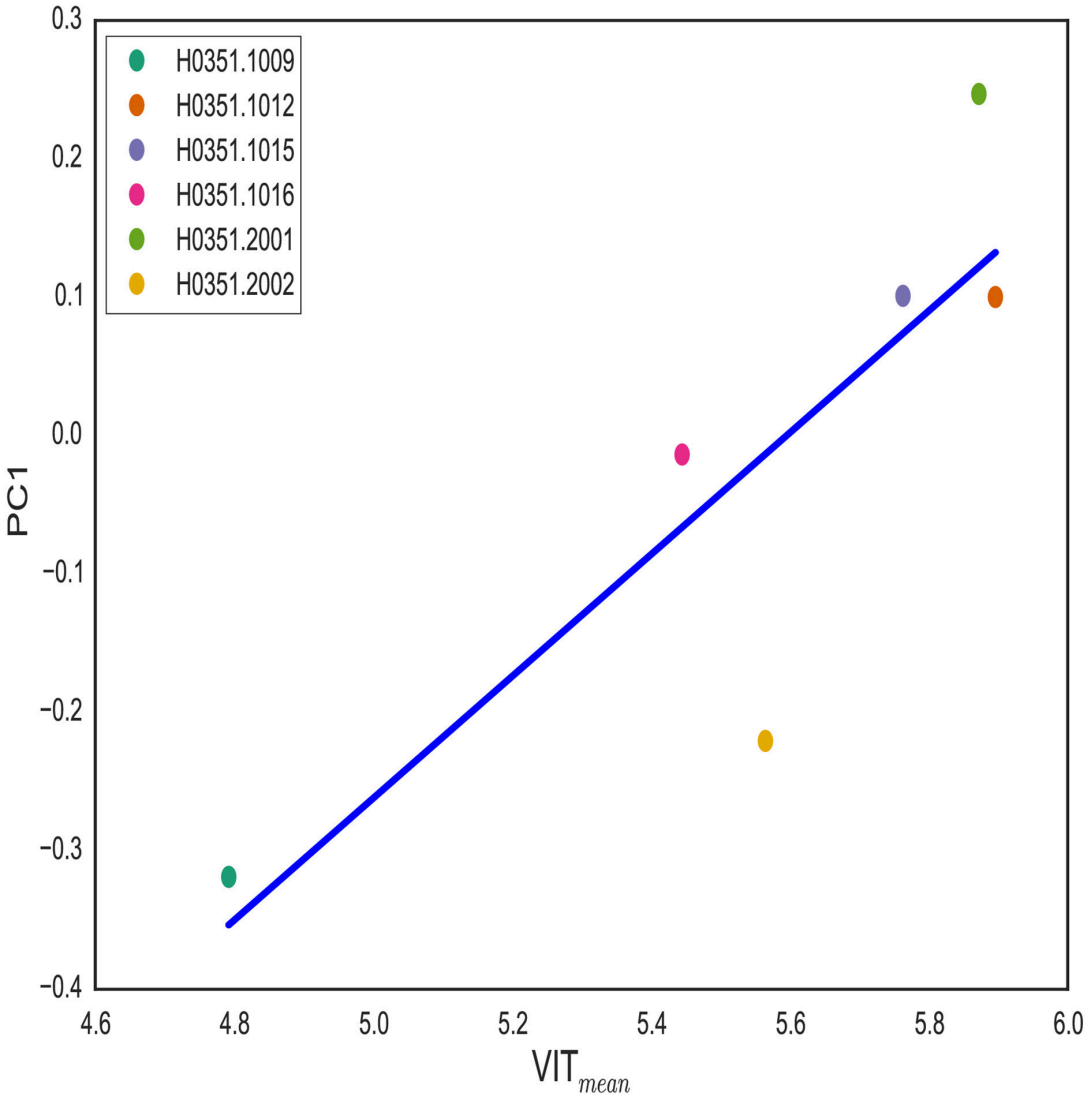
**VIT expression is associated with PC1**. VIT gene expression in left anterior cingulate cortex is correlated with PC1 score (*P* = 0.03). For each individual, log_2_ VIT expression from all samples taken from left anterior cingulate cortex is averaged. PC1 score for each individual is computed by projecting asymmetry scores of each individual onto PC1 from CN group.

The rs11691187 is in the first intron of the *VIT* gene. We hypothesized that *VIT* gene expression is associated with brain asymmetry along PC1. In order to test this hypothesis, we used publicly available Allen Human Brain Atlas (AHBA). We obtained normalized log2 *VIT* expression of samples taken from left anterior cingulate cortex (both caudal and rostral parts) (see Methods) as this region displayed highest weight in PC1 loadings. We used both median and mean *VIT* gene expression for samples taken from each individual. T1-weighted image for each subject was obtained and analyzed to get cortical surface areas of 34 regions as described previously. *VIT* expression showed positive correlation with computed PC1 scores (see Methods) (*P* = 0.03 for mean, *P* = 0.05 for median) (Figure 5). This finding further supports the role of *VIT* in brain asymmetry variation.

## Discussion

In this study, we aimed to find genetic variants associated with a subset of brain asymmetry variation across individuals. To do so, we computed cortical surface area asymmetry for 34 regions throughout cerebral cortex. We then used PCA to find an axis that captures the most brain asymmetry variation among individuals. The first principal component (PC1) explained around 13% of brain asymmetry variation. We replicated PC1 in a separate dataset and showed that PC1 was highly stable across time and groups. We hypothesized that genetic factors account for part of the variation along PC1 axis. We performed a GWAS to find genetic variants that are associated with the PC1 scores and identified one SNP (rs11691187) that showed strong association (*P* = 5.69e-08). We replicated this finding in a separate cohort with a combined *P*-value that was genome-wide significant (*P* = 2.40e-08).

Biological mechanisms underlying brain asymmetry are largely unknown. In 1964, Mariane Annet (Annett, 1964) proposed Right-shift theory which holds that a single gene with 2 alleles control brain asymmetry and cerebral dominance. Although no gene has been identified, prior studies have proposed genetic loci that influence brain asymmetry. Deviations of brain asymmetry in sex chromosome aneuploidies have led some researchers to candidate loci within sex chromosomes (Rezaie et al., 2009). In particular, protocadherin 11X/Y (PCDH11X/Y) within Xq21.3/Yp11.2 human specific homology region has been claimed as a potential candidate for brain asymmetry (Priddle and Crow, 2013). PCDH11X/Y encodes cell adhesion molecules and has been subject to accelerated evolution following the separation of the human and chimpanzee lineages (Williams et al., 2006). Several other genes including FOXP2(Ocklenburg et al., 2013b), LRRTM1(Francks et al., 2007), AR (Medland et al., 2005), CCKAR (Ocklenburg et al., 2013a), and GRIN2B (Ocklenburg et al., 2011) have been proposed as potential candidates; however, they require further validation. On the other hand, other hypotheses have recognized the multifactorial nature of brain asymmetry. In their influential hypothesis, Geschwind and Galaburda (1985) claimed that the intrauterine circulating levels of testosterone contribute to brain asymmetry. Recent studies using brain intrinsic activity at rest have shown that brain asymmetry can be decomposed into several independent factors (Liu et al., 2009). Thus it has been argued that one single gene or environmental factor can only explain a part of asymmetry variation among individuals. Here, we have extended this idea to anatomical brain asymmetry variation using PCA to decompose structural brain asymmetry into orthogonal components. The use of this technique enabled us to capture correlated variation in asymmetry, which in turn increased the power to detect genetic variants associated with these correlated variations.

PCA revealed that several components contribute to cortical surface area asymmetry variation. The first few components are heavily loaded on a few specific areas (See Supplementary Figure 3). These components are also generally stable over time in healthy subjects (Supplementary Figure 5). We focused our analysis on the first principal component. We did so as the first principal component captures the highest variation and is stable across time and groups. Among the 34 regions, cACC, and rACC showed the largest weights in PC1. Several studies have demonstrated structural and functional asymmetries of ACC (Gong et al., 2005; Huster et al., 2007; Yan et al., 2009; Wang et al., 2013). In line with previous studies we found that cACC and rACC have rightward and leftward surface area asymmetries, respectively (Koelkebeck et al., 2014). Altered asymmetry of ACC has been noted in subjects at high genetic risk for psychosis and schizophrenia (Park et al., 2013). Understanding genetic factors that modulate ACC brain asymmetry can shed light on schizophrenia and other neuropsychological disorders as well.

The SNP that was highly associated with PC1 in this study is located in the first intron of *VIT*. We explored the association of *VIT* gene expression with PC1 in six donors of AHBA. We found a significant association between *VIT* gene expression in left anterior cingulate cortex and extent of brain asymmetry along PC1. However, given the small sample size in AHBA, future study with a larger sample size is necessary to confirm these findings.

*VIT* is widely expressed throughout cerebral cortex (Allen Institute for Brain Science^1^). *VIT* encodes an extracellular matrix (ECM) protein, vitrin. Vitrin was originally isolated from the vitreous of bovine eye (Mayne et al., 1999) and contains a single LCCL domain (named after Limulus factor C, Coch-5b2 and Lgl1 protein) followed by two von Willebrand A (VWA) domains. Majority of VWA-containing proteins are extracellular and participate in biological processes such as cell adhesion and migration (Colombatti et al., 1993; Whittaker and Hynes, 2002). Also vitrin displays high homology and structural similarity to Akhirin and cochlin (Ahsan et al., 2005). Both Akhirin and cochlin are involved in neural development and extracellular matrix integrity (Zhang et al., 2013; Bae et al., 2014; Abdulhaleem et al., 2015). These structural similarities suggest the possible role of vitrin in matrix assembly, cell adhesion, and migration, processes that are crucial for neural development. Furthermore, *VIT* is expressed highly in lateral and caudal ganglionic eminences during neural development in humans (Allen Institute for Brain Science^1^). These structures are responsible for neural migration (Nadarajah and Parnavelas, 2002), which suggests possible role of vitrin in neural development. Prior studies have suggested that individual differences in brain asymmetry probably arise from factors that exert their functions early in development (Sun et al., 2005; Francks, 2015). Moreover, cell adhesion molecules have been proposed as potential candidates for brain asymmetry (Priddle and Crow, 2013; Francks, 2015). Further, studies are needed to elucidate the mechanisms by which *VIT* influences brain asymmetry.

The present study has limitations that should be addressed in future studies. In our study, we merged three groups of AD, LMCI, and CN for the interest of increased sample size. Although we showed that PC1 is a stable component across groups and time, which allowed us to combine three groups for subsequent GWAS study, it would be appropriate to test this variant in larger normal sample sizes. Furthermore, *VIT* gene expression showed significant correlation with brain asymmetry along the PC1 axis. Due to small sample size in the Allen Human Brain Atlas, this result should be validated in future studies.

In conclusion, we have found a common genetic variant that is associated with cortical surface area asymmetry variation among individuals. This variant resides within *VIT* gene. *VIT* is known to play a role in neural development. Our findings provide evidence that genetic variants can modulate brain asymmetry.

## Author contributions

ST designed the experiment, analyzed the data and wrote the paper. MV helped in designing the experiment, intepreting the results, and writing the paper. PK helped in neuroimaging data analysis and interpreting the results. MA helped in GWAS interpretation and analysis. AA helped in designing the experiment and writing the paper.

### Conflict of interest statement

The authors declare that the research was conducted in the absence of any commercial or financial relationships that could be construed as a potential conflict of interest.

## Footnotes

^1^*Allen Institute for Brain Science Allen Human Brain Atlas*. Available online at: http://human.brain-map.org.

## References

[B1] Abdulhaleem M. F. Song X. Kawano R. Uezono N. Ito A. Ahmed G.. (2015). Akhirin regulates the proliferation and differentiation of neural stem cells in intact and injured mouse spinal cord. Dev. Neurobiol. 75, 494–504. 10.1002/dneu.22238 25331329

[B2] Ahsan M. Ohta K. Kuriyama S. Tanaka H. (2005). Novel soluble molecule, Akhirin, is expressed in the embryonic chick eyes and exhibits heterophilic cell-adhesion activity. Dev. Dyn. 233, 95–104. 10.1002/dvdy.20303 15765510

[B3] Allen Institute for Brain Science (2013). Microarray Data Normalization. Available online at: http://help.brain-map.org/download/attachments/2818165/Normalization_WhitePaper.pdf?version=1&modificationDate=1361836502191

[B4] Annett M. (1964). A Model of the inheritance of handedness and cerebral dominance. Nature 204, 59–60. 10.1038/204059a0 14240116

[B5] Bae S. H. Robertson N. G. Cho H. J. Morton C. C. Jung da J. Baek J. I.. (2014). Identification of pathogenic mechanisms of COCH mutations, abolished cochlin secretion, and intracellular aggregate formation: genotype-phenotype correlations in DFNA9 deafness and vestibular disorder. Hum. Mutat. 35, 1506–1513. 10.1002/humu.22701 25230692 PMC4373469

[B6] Bakken T. E. Roddey J. C. Djurovic S. Akshoomoff N. Amaral D. G. Bloss C. S.. (2012). Association of common genetic variants in GPCPD1 with scaling of visual cortical surface area in humans. Proc. Natl. Acad. Sci. U.S.A. 109, 3985–3990. 10.1073/pnas.1105829109 22343285 PMC3309762

[B7] Bishop D. V. (2013). Cerebral asymmetry and language development: cause, correlate, or consequence? Science 340:1230531. 10.1126/science.1230531 23766329 PMC4031634

[B8] Cai D. C. Fonteijn H. Guadalupe T. Zwiers M. Wittfeld K. Teumer A.. (2014). A genome-wide search for quantitative trait loci affecting the cortical surface area and thickness of Heschl's gyrus. Genes Brain Behav. 13, 675–685. 10.1111/gbb.12157 25130324

[B9] Callicott J. H. Straub R. E. Pezawas L. Egan M. F. Mattay V. S. Hariri A. R.. (2005). Variation in DISC1 affects hippocampal structure and function and increases risk for schizophrenia. Proc. Natl. Acad. Sci. U.S.A. 102, 8627–8632. 10.1073/pnas.0500515102 15939883 PMC1143583

[B10] Colombatti A. Bonaldo P. Doliana R. (1993). Type A modules: interacting domains found in several non-fibrillar collagens and in other extracellular matrix proteins. Matrix 13, 297–306. 10.1016/S0934-8832(11)80025-9 8412987

[B11] Corballis M. C. (2014). Left brain, right brain: facts and fantasies. PLoS Biol. 12:e1001767. 10.1371/journal.pbio.1001767 24465175 PMC3897366

[B12] de Bakker P. I. Ferreira M. A. Jia X. Neale B. M. Raychaudhuri S. Voight B. F. (2008). Practical aspects of imputation-driven meta-analysis of genome-wide association studies. Hum. Mol. Genet. 17, R122–R128. 10.1093/hmg/ddn288 18852200 PMC2782358

[B13] Desikan R. S. Ségonne F. Fischl B. Quinn B. T. Dickerson B. C. Blacker D.. (2006). An automated labeling system for subdividing the human cerebral cortex on MRI scans into gyral based regions of interest. Neuroimage 31, 968–980. 10.1016/j.neuroimage.2006.01.021 16530430

[B14] Devlin B. Roeder K. Wasserman L. (2001). Genomic control, a new approach to genetic-based association studies. Theor. Popul. Biol. 60, 155–166. 10.1006/tpbi.2001.1542 11855950

[B15] Eyler L. T. Vuoksimaa E. Panizzon M. S. Fennema-Notestine C. Neale M. C. Chen C. H.. (2014). Conceptual and data-based investigation of genetic influences and brain asymmetry: a twin study of multiple structural phenotypes. J. Cogn. Neurosci. 26, 1100–1117. 10.1162/jocn_a_00531 24283492 PMC3999438

[B16] Francks C. (2015). Exploring human brain lateralization with molecular genetics and genomics. Ann. N.Y. Acad. Sci. 1359, 1–13. 10.1111/nyas.12770 25950729

[B17] Francks C. Maegawa S. Laurén J. Abrahams B. S. Velayos-Baeza A. Medland S. E.. (2007). LRRTM1 on chromosome 2p12 is a maternally suppressed gene that is associated paternally with handedness and schizophrenia. Mol. Psychiatry 12, 1129–1139, 1057. 10.1038/sj.mp.4002053 17667961 PMC2990633

[B18] Geschwind N. Galaburda A. M. (1985). Cerebral lateralization. biological mechanisms, associations, and pathology: III. A hypothesis and a program for research. Arch. Neurol. 42, 634–654. 10.1001/archneur.1985.04060070024012 3874617

[B19] Gong G. Jiang T. Zhu C. Zang Y. Wang F. Xie S.. (2005). Asymmetry analysis of cingulum based on scale-invariant parameterization by diffusion tensor imaging. Hum. Brain Mapp. 24, 92–98. 10.1002/hbm.20072 15455461 PMC6871701

[B20] Guadalupe T. Zwiers M. P. Wittfeld K. Teumer A. Vasquez A. A. Hoogman M.. (2015). Asymmetry within and around the human planum temporale is sexually dimorphic and influenced by genes involved in steroid hormone receptor activity. Cortex 62, 41–55. 10.1016/j.cortex.2014.07.015 25239853

[B21] Habeck C. G. (2010). Basics of multivariate analysis in neuroimaging data. J. Vis. Exp. 10.3791/1988. [Epub ahead of print]. 20689509 PMC3074457

[B22] Hawrylycz M. J. Lein E. S. Guillozet-Bongaarts A. L. Shen E. H. Ng L. Miller J. A.. (2012). An anatomically comprehensive atlas of the adult human brain transcriptome. Nature 489, 391–399. 10.1038/nature11405 22996553 PMC4243026

[B23] Huster R. J. Westerhausen R. Kreuder F. Schweiger E. Wittling W. (2007). Morphologic asymmetry of the human anterior cingulate cortex. Neuroimage 34, 888–895. 10.1016/j.neuroimage.2006.10.023 17161625

[B24] Hutsler J. J. Loftus W. C. Gazzaniga M. S. (1998). Individual variation of cortical surface area asymmetries. Cereb. Cortex 8, 11–17. 10.1093/cercor/8.1.11 9510381

[B25] Jahanshad N. Lee A. D. Barysheva M. McMahon K. L. de Zubicaray G. I. Martin N. G.. (2010). Genetic influences on brain asymmetry: a DTI study of 374 twins and siblings. Neuroimage 52, 455–469. 10.1016/j.neuroimage.2010.04.236 20430102 PMC3086641

[B26] Jahanshad N. Rajagopalan P. Hua X. Hibar D. P. Nir T. M. Toga A. W.. (2013). Genome-wide scan of healthy human connectome discovers SPON1 gene variant influencing dementia severity. Proc. Natl. Acad. Sci. U.S.A. 110, 4768–4773. 10.1073/pnas.1216206110 23471985 PMC3606977

[B27] Koelkebeck K. Miyata J. Kubota M. Kohl W. Son S. Fukuyama H.. (2014). The contribution of cortical thickness and surface area to gray matter asymmetries in the healthy human brain. Hum. Brain Mapp. 35, 6011–6022. 10.1002/hbm.22601 25082171 PMC6869478

[B28] Liu H. Stufflebeam S. M. Sepulcre J. Hedden T. Buckner R. L. (2009). Evidence from intrinsic activity that asymmetry of the human brain is controlled by multiple factors. Proc. Natl. Acad. Sci. U.S.A. 106, 20499–20503. 10.1073/pnas.0908073106 19918055 PMC2777963

[B29] Luders E. Narr K. L. Thompson P. M. Rex D. E. Jancke L. Toga A. W. (2006). Hemispheric asymmetries in cortical thickness. Cereb. Cortex 16, 1232–1238. 10.1093/cercor/bhj064 16267139

[B30] Lyttelton O. C. Karama S. Ad-Dab'bagh Y. Zatorre R. J. Carbonell F. Worsley K.. (2009). Positional and surface area asymmetry of the human cerebral cortex. Neuroimage 46, 895–903. 10.1016/j.neuroimage.2009.03.063 19345735

[B31] Marenco S. Radulescu E. (2010). Imaging genetics of structural brain connectivity and neural integrity markers. Neuroimage 53, 848–856. 10.1016/j.neuroimage.2009.11.030 19932755 PMC2889028

[B32] Mayne R. Ren Z. X. Liu J. Cook T. Carson M. Narayana S. (1999). VIT-1: the second member of a new branch of the von Willebrand factor A domain superfamily. Biochem. Soc. Trans. 27, 832–835. 10.1042/bst0270832 10830112

[B33] Medland S. E. Duffy D. L. Spurdle A. B. Wright M. J. Geffen G. M. Montgomery G. W.. (2005). Opposite effects of androgen receptor CAG repeat length on increased risk of left-handedness in males and females. Behav. Genet. 35, 735–744. 10.1007/s10519-005-6187-3 16273319

[B34] Meyer M. Liem F. Hirsiger S. Jäncke L. Hänggi J. (2014). Cortical surface area and cortical thickness demonstrate differential structural asymmetry in auditory-related areas of the human cortex. Cereb. Cortex 24, 2541–2552. 10.1093/cercor/bht094 23645712

[B35] Nadarajah B. Parnavelas J. G. (2002). Modes of neuronal migration in the developing cerebral cortex. Nat. Rev. Neurosci. 3, 423–432. 10.1038/nrn845 12042877

[B36] Ocklenburg S. Arning L. Gerding W. M. Epplen J. T. Güntürkün O. Beste C. (2013a). Cholecystokinin a receptor (CCKAR) gene variation is associated with language lateralization. PLoS ONE 8:e53643. 10.1371/journal.pone.0053643 23341962 PMC3544920

[B37] Ocklenburg S. Arning L. Gerding W. M. Epplen J. T. Güntürkün O. Beste C. (2013b). FOXP2 variation modulates functional hemispheric asymmetries for speech perception. Brain Lang. 126, 279–284. 10.1016/j.bandl.2013.07.001 23911943

[B38] Ocklenburg S. Arning L. Hahn C. Gerding W. M. Epplen J. T. Güntürkün O.. (2011). Variation in the NMDA receptor 2B subunit gene GRIN2B is associated with differential language lateralization. Behav. Brain Res. 225, 284–289. 10.1016/j.bbr.2011.07.042 21827795

[B39] Park H. Y. Hwang J. Y. Jung W. H. Shin N. Y. Shim G. Jang J. H.. (2013). Altered asymmetry of the anterior cingulate cortex in subjects at genetic high risk for psychosis. Schizophr. Res. 150, 512–518. 10.1016/j.schres.2013.08.027 24035404

[B40] Potkin S. G. Guffanti G. Lakatos A. Turner J. A. Kruggel F. Fallon J. H.. (2009). Hippocampal atrophy as a quantitative trait in a genome-wide association study identifying novel susceptibility genes for Alzheimer's disease. PLoS ONE 4:e6501. 10.1371/journal.pone.0006501 19668339 PMC2719581

[B41] Priddle T. H. Crow T. J. (2013). The protocadherin 11X/Y (PCDH11X/Y) gene pair as determinant of cerebral asymmetry in modern Homo sapiens. Ann. N.Y. Acad. Sci. 1288, 36–47. 10.1111/nyas.12042 23600975 PMC3752934

[B42] Pruim R. J. Welch R. P. Sanna S. Teslovich T. M. Chines P. S. Gliedt T. P.. (2010). LocusZoom: regional visualization of genome-wide association scan results. Bioinformatics 26, 2336–2337. 10.1093/bioinformatics/btq419 20634204 PMC2935401

[B43] Purcell S. Neale B. Todd-Brown K. Thomas L. Ferreira M. A. Bender D.. (2007). PLINK: a tool set for whole-genome association and population-based linkage analyses. Am. J. Hum. Genet. 81, 559–575. 10.1086/519795 17701901 PMC1950838

[B44] Ramanan V. K. Nho K. Shen L. Risacher S. L. Kim S. McDonald B. C.. (2015). FASTKD2 is associated with memory and hippocampal structure in older adults. Mol. Psychiatry 20, 1197–1204. 10.1038/mp.2014.142 25385369 PMC4427556

[B45] Rezaie R. Daly E. M. Cutter W. J. Murphy D. G. Robertson D. M. DeLisi L. E.. (2009). The influence of sex chromosome aneuploidy on brain asymmetry. Am. J. Med. Genet. B Neuropsychiatr. Genet. 150B, 74–85. 10.1002/ajmg.b.30772 18454450

[B46] Roussotte F. F. Jahanshad N. Hibar D. P. Sowell E. R. Kohannim O. Barysheva M.. (2014). A commonly carried genetic variant in the delta opioid receptor gene, OPRD1, is associated with smaller regional brain volumes: replication in elderly and young populations. Hum. Brain Mapp. 35, 1226–1236. 10.1002/hbm.22247 23427138 PMC4046708

[B47] Satagopan J. M. Venkatraman E. S. Begg C. B. (2004). Two-stage designs for gene-disease association studies with sample size constraints. Biometrics 60, 589–597. 10.1111/j.0006-341X.2004.00207.x 15339280 PMC8985053

[B48] Skol A. D. Scott L. J. Abecasis G. R. Boehnke M. (2006). Joint analysis is more efficient than replication-based analysis for two-stage genome-wide association studies. Nat. Genet. 38, 209–213. 10.1038/ng1706 16415888

[B49] Stein J. L. Medland S. E. Vasquez A. A. Hibar D. P. Senstad R. E. Winkler A. M.. (2012). Identification of common variants associated with human hippocampal and intracranial volumes. Nat. Genet. 44, 552–561. 10.1038/ng.2250 22504417 PMC3635491

[B50] Sun T. Patoine C. Abu-Khalil A. Visvader J. Sum E. Cherry T. J.. (2005). Early asymmetry of gene transcription in embryonic human left and right cerebral cortex. Science 308, 1794–1798. 10.1126/science.1110324 15894532 PMC2756725

[B51] Takaya S. Kuperberg G. R. Liu H. Greve D. N. Makris N. Stufflebeam S. M. (2015). Asymmetric projections of the arcuate fasciculus to the temporal cortex underlie lateralized language function in the human brain. Front. Neuroanat. 9:119. 10.3389/fnana.2015.00119 26441551 PMC4569731

[B52] Wang J. Liu D. Q. Zhang H. Zhu W. X. Dong Z. Y. Zang Y. F. (2013). Asymmetry of the dorsal anterior cingulate cortex: evidences from multiple modalities of MRI. Neuroinformatics 11, 149–157. 10.1007/s12021-012-9167-9 23055047

[B54] Whittaker C. A. Hynes R. O. (2002). Distribution and evolution of von Willebrand/integrin A domains: widely dispersed domains with roles in cell adhesion and elsewhere. Mol. Biol. Cell 13, 3369–3387. 10.1091/mbc.E02-05-0259 12388743 PMC129952

[B53] Williams N. A. Close J. P. Giouzeli M. Crow T. J. (2006). Accelerated evolution of Protocadherin11X/Y: a candidate gene-pair for cerebral asymmetry and language. Am. J. Med. Genet. B Neuropsychiatr. Genet. 141B, 623–633. 10.1002/ajmg.b.30357 16874762

[B55] Yan H. Zuo X. N. Wang D. Wang J. Zhu C. Milham M. P.. (2009). Hemispheric asymmetry in cognitive division of anterior cingulate cortex: a resting-state functional connectivity study. Neuroimage 47, 1579–1589. 10.1016/j.neuroimage.2009.05.080 19501172

[B56] Zhang J. Fei T. Li Z. Zhu G. Wang L. Chen Y. G. (2013). BMP induces cochlin expression to facilitate self-renewal and suppress neural differentiation of mouse embryonic stem cells. J. Biol. Chem. 288, 8053–8060. 10.1074/jbc.M112.433995 23344953 PMC3605624

