# Supplementary material for: Common Genetic Variant in VIT Is Associated with Human Brain Asymmetry
Source: Front Hum Neurosci. 2016 May 24;10:236. doi: 10.3389/fnhum.2016.00236 (PMC4877381; doi:10.3389/fnhum.2016.00236)
Supplement: Supplementary file 1 [file Table1.PDF]

Area	F-test	P	TukeyHSD P
**Banksst**	0.118	0.889	-
**Caudal anterior cingulate**	0.424	0.655	-
**Caudal middle frontal**	2.565	0.0776	-
**Cuneus**	0.415	0.66	-
**Entorhinal**	0.472	0.624	-
**Frontal pole**	3.096	0.0458*	HC-ALZ: 0.048
**Fusiform**	0.653	0.521	-
**Inferior parietal**	1.016	0.362	-
**Inferior temporal**	0.095	0.909	-
**Insula**	0.063	0.939	-
**Isthmus cingulate**	1.849	0.158	-
**Lateral occipital**	0.097	0.908	-
**Lateral orbitofrontal**	0.621	0.538	-
**Lingual**	1.3	0.273	-
**Medial orbitofrontal**	1.44	0.238	-
**Middle temporal**	0.908	0.404	-
**Paracentral**	2.21	0.11	-
**Parahippocampal**	0.801	0.449	-
**Pars opercularis**	2.483	0.0843	-
**Pars orbitalis**	3.194	0.0416*	LMCI-ALZ: 0.039
**Pars triangularis**	0.838	0.433	-
**Pericalcarine**	0.604	0.547	-
**Postcentral**	0.107	0.898	-
**Posterior cingulate**	1.091	0.336	-
**Precentral**	0.153	0.858	-
**Precuneus**	0.942	0.39	-
**Rostral anterior cingulate**	1.991	0.137	-
**Rostral middle frontal**	0.091	0.913	-
**Superior frontal**	0.581	0.56	-
**Superior parietal**	0.336	0.715	-
**Superior temporal**	0.435	0.647	-
**Supramarginal**	0.517	0.596	-
**Temporal pole**	0.823	0.439	-
**Transverse temporal**	0.901	0.406	-

Supplementary Table 1: ANOVA test to compare brain asymmetry across HC, LMCI and AD. Two areas passed P-value <0.05 but none passed Bonferroni correction (0.05/34=0.0015).
