# Supplementary material for: Common Genetic Variant in VIT Is Associated with Human Brain Asymmetry
Source: Front Hum Neurosci. 2016 May 24;10:236. doi: 10.3389/fnhum.2016.00236 (PMC4877381; doi:10.3389/fnhum.2016.00236)
Supplement: Supplementary file 2 [file Image1.PDF]

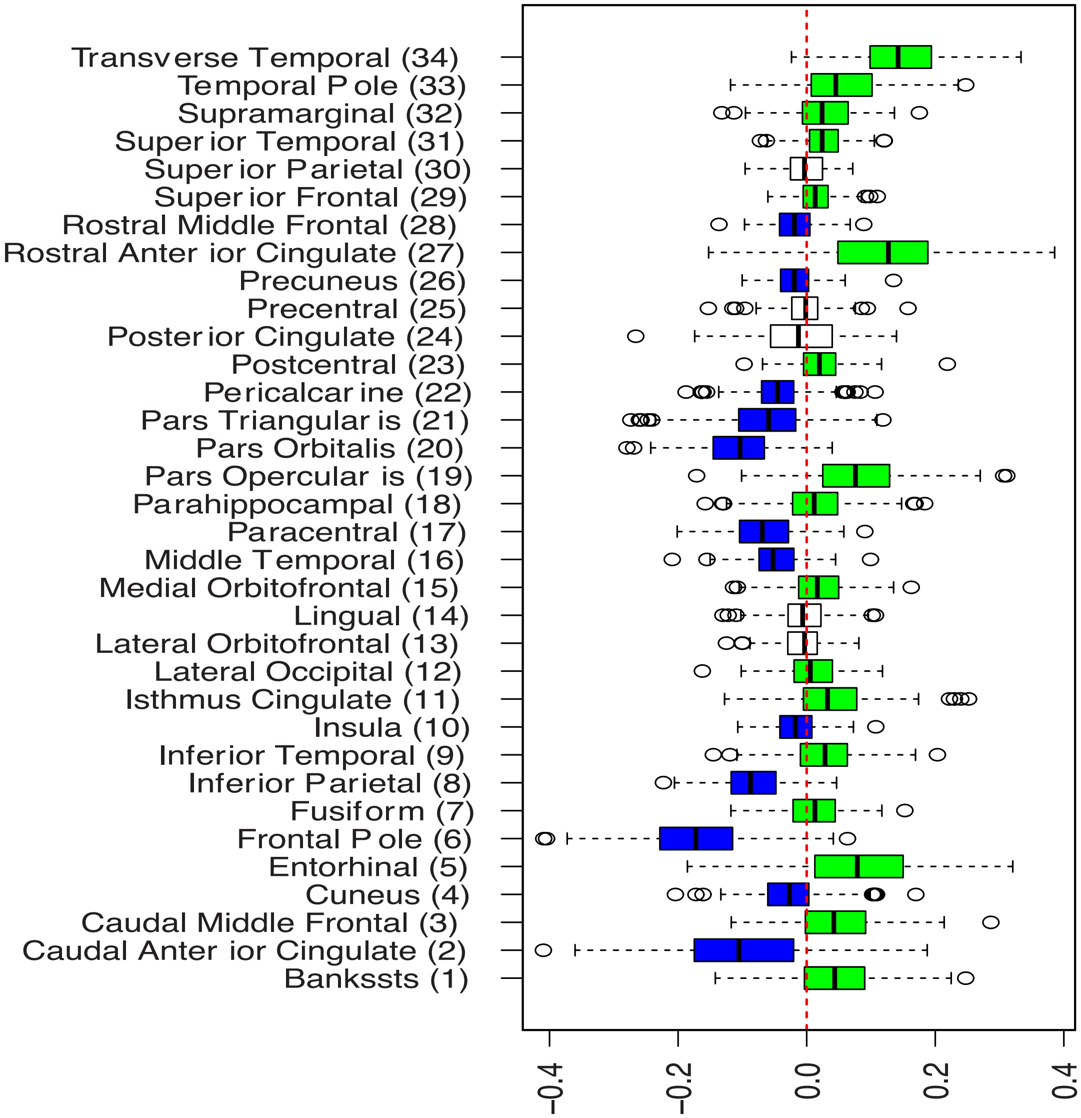

Supplementary Figure 1: Cortical surface brain asymmetry variation for 34 cortical regions in CN group. Positive indices imply left > right asymmetry. Green boxplots and blue boxplots show significant leftward or rightward asymmetry, respectively. (One sample t-test with mean zero. A stringent threshold of 0.001 was set due to Bonferroni correction (0.05/34)). White boxplots show areas that did not pass threshold. (Bankssts: Posterior banks of superior temporal sulcus)
