# Supplementary material for: Common Genetic Variant in VIT Is Associated with Human Brain Asymmetry
Source: Front Hum Neurosci. 2016 May 24;10:236. doi: 10.3389/fnhum.2016.00236 (PMC4877381; doi:10.3389/fnhum.2016.00236)
Supplement: Supplementary file 3 [file Image2.PDF]

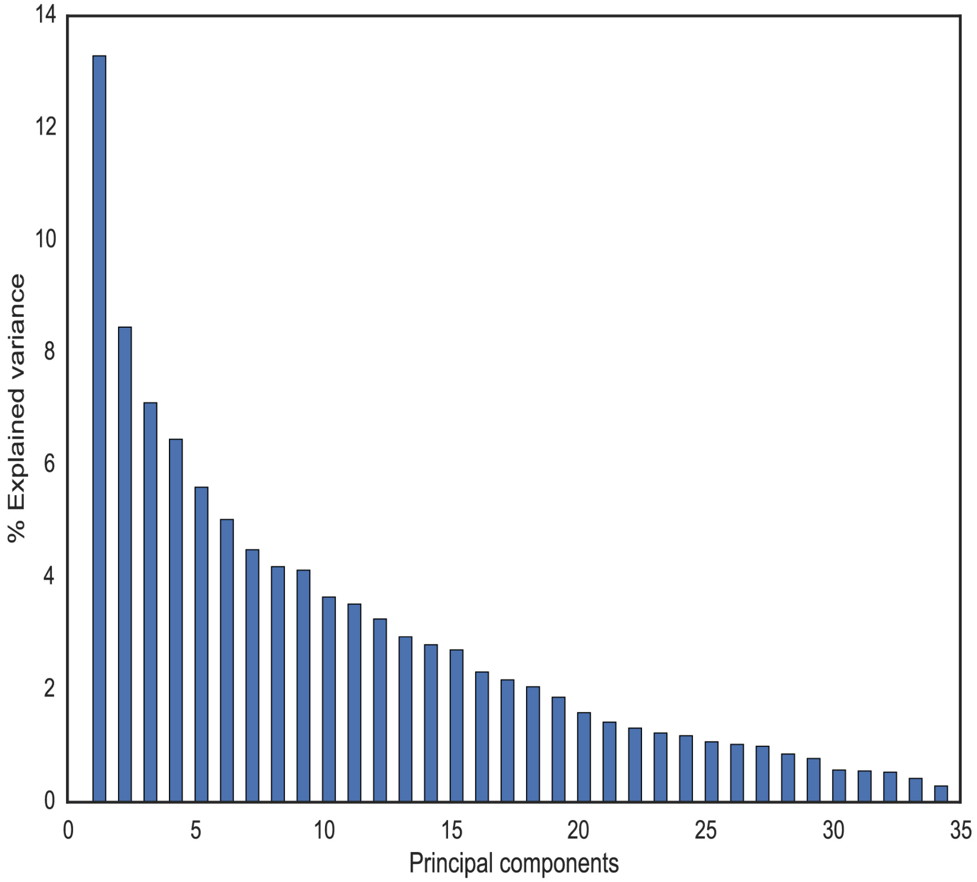

Supplementary Figure 2: Principal component analysis. Explained variance percentage for each component. The first principal component explains 13% of brain asymmetry variation.
