# Supplementary material for: Common Genetic Variant in VIT Is Associated with Human Brain Asymmetry
Source: Front Hum Neurosci. 2016 May 24;10:236. doi: 10.3389/fnhum.2016.00236 (PMC4877381; doi:10.3389/fnhum.2016.00236)
Supplement: Supplementary file 5 [file Image4.PDF]

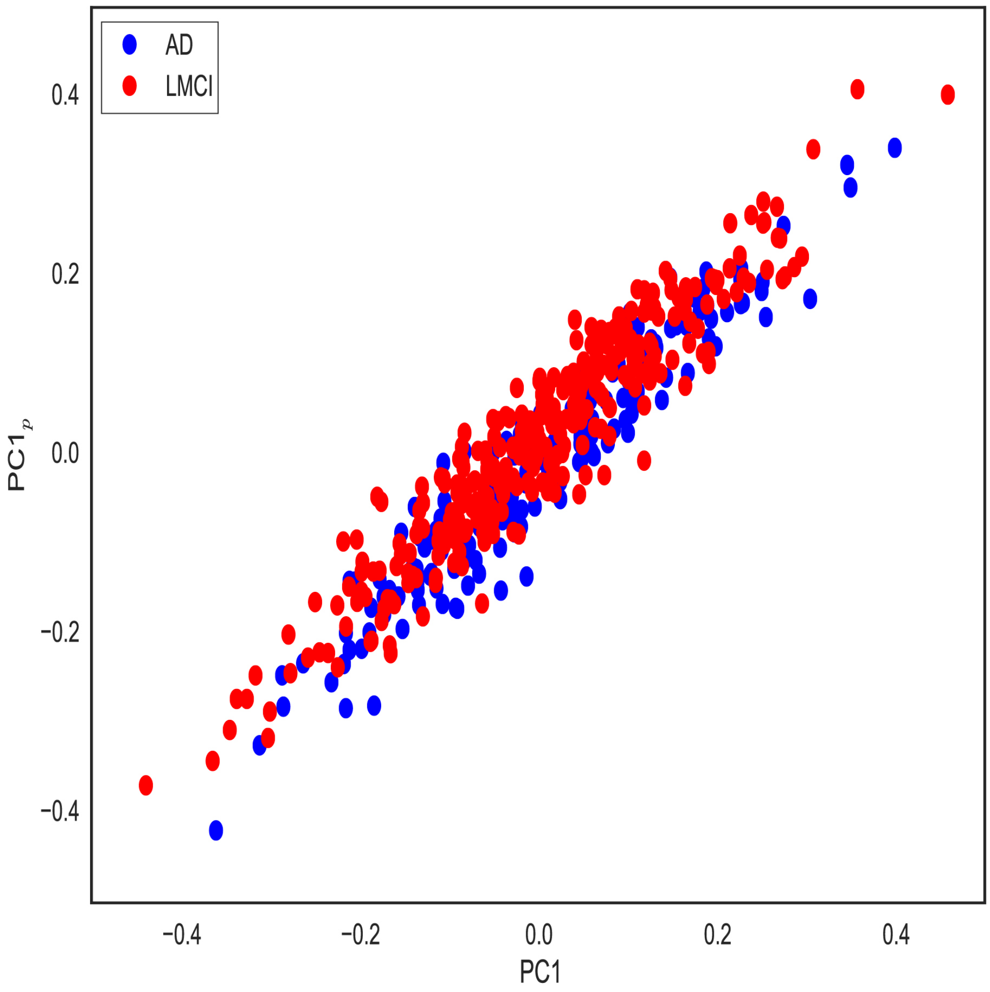

Supplementary Figure 4: Projection of AD and LMCI asymmetry scores on PC1 axis of HC. The x-axis is the PC1 score from AD and LMCI original PCA and $PC1_p$ is scores from projection onto HC PC1.
