# Supplementary material for: Common Genetic Variant in VIT Is Associated with Human Brain Asymmetry
Source: Front Hum Neurosci. 2016 May 24;10:236. doi: 10.3389/fnhum.2016.00236 (PMC4877381; doi:10.3389/fnhum.2016.00236)
Supplement: Supplementary file 6 [file Image5.PDF]

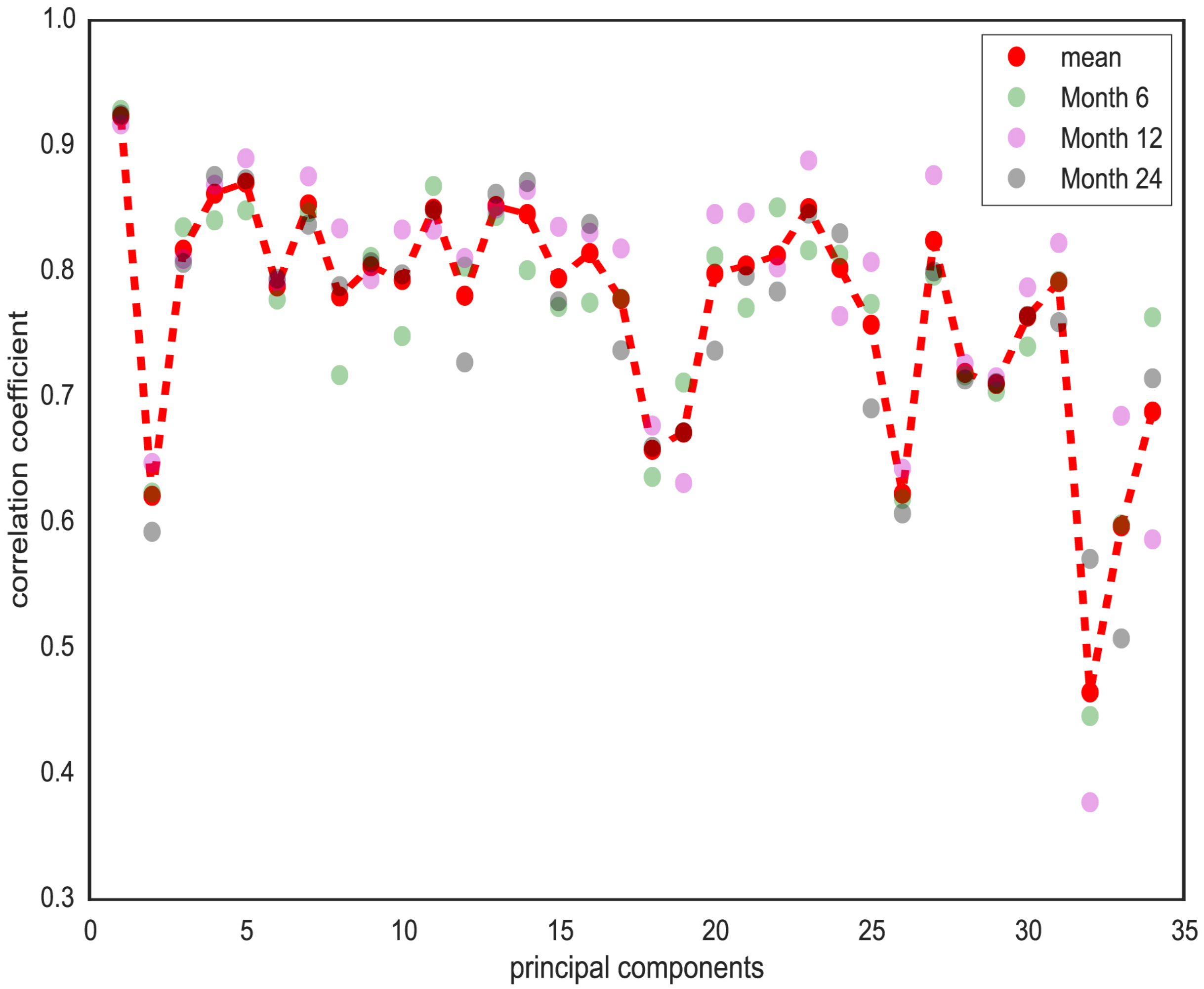

Supplementary Figure 5: Stability of principal components over time. The y-axis is the correlation coefficient between each principal component's loadings at screening time with loadings at Month 6, Month 12 and Month 24. As the plot shows, most principal components are stable over time.
