# Supplementary material for: Common Genetic Variant in VIT Is Associated with Human Brain Asymmetry
Source: Front Hum Neurosci. 2016 May 24;10:236. doi: 10.3389/fnhum.2016.00236 (PMC4877381; doi:10.3389/fnhum.2016.00236)
Supplement: Supplementary file 7 [file Image6.PDF]

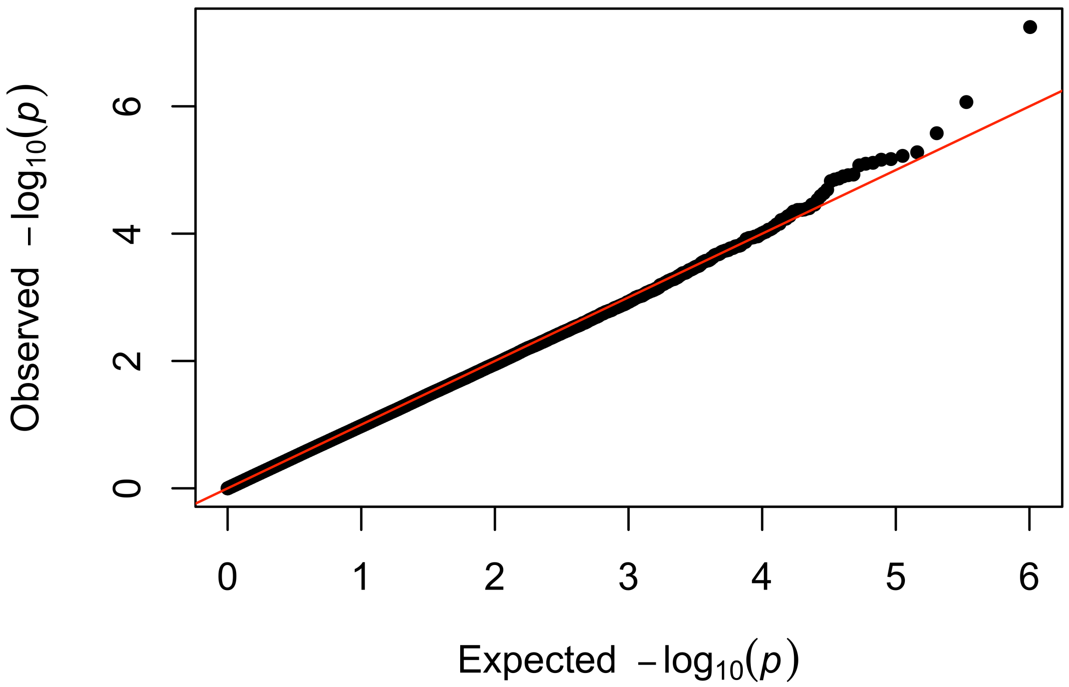

Supplementary Figure 6: GWAS QQ plot. Q-Q plot of the distribution of P-values shows that the association statistics are approximately Normal. Genomic inflation ($\boldsymbol{\lambda}_{GC}$) was 1.
